# Interstate Comity in Dental Legislation

**Published:** 1900-01

**Authors:** Rodrigues Ottolengui

**Affiliations:** New York.


AMERICAN JOURNAL

—OF—

DENTAL SCIENCE.

VOL. XXXIII. BALTIMORE, JANUARY, 1900. No. 9.

### Selections.

ARTICLE I.

### INTERSTATE COMITY IN DENTAL LEGISLATION.

RODRIGUES OTTOLENGUI, M. D. S., NEW YORK.

**Report as correspondent read at the annual meeting of New York State Dental Society, May, 1899, and here published by courteous permission of the Editor of the "Cosmos."**

Mr. President and Gentlemen :—In seeking a subject about which to correspond with members of the profession, in order to obtain a basis for the usual Correspondent's report, it has seemed to me that the one subject which, at present, interests the larger number of dentists throughout the country, and the one which is in most need of discussion and final settlement, is the question—"How best to unify the various State dental laws throughout the United States ?"

The following is a copy of the letter which I sent to the secretaries of all the State Examining Boards in the country:

"My Dear Doctor:—As Correspondent of the New York State Society, it is my duty to annually present a report upon some feature of dental practice of vital interest to the whole profession. At the present time there seems nothing which will arouse more universal interest than a discussion of some method by which a uniform standard of education, or more especially of the granting of licenses could be arranged throughout the States.

I send you herewith a copy of *Items of Interest* for February, in which you will find a paper on the subject by Prof. Kirk, and also a discussion. I would ask you also to read my editorlal.

Having done this, I would like you to send me, with permission to report it to my society, your own opinion, and, if possible, the opinion of your entire board, it it could be obtained at a meeting, as to the feasibility of either of the following methods.

First, the one suggested in the editorial—That applicants for licenses should be required to present the papers upon which they were examined at college when obtaining their diploma. The State Board, upon being satisfied through these original examination papers, that the applicant is worthy, to give him a license to practice; and, secondly, to accept a license of this character granted in similar manner by any other State.

Second Proposition—For all State Boards, members of the National Association of Dental Examiners, to make use of identical examination papers, the same being prepared by a committee of the national body; licenses granted upon such examination papers to be interchangeable among the states represented in the National Association.

Is it not a fact that without appealing to the legislature of your state, your body is privileged to consider either of the above methods a satisfactory examination of the candidate? This is to say, is not the examination letf to the determination of your Board? In which case, you will be at liberty to carry out either of the above propositions.

Lastly, in case neither of these would seem practical in your state, have you any alternate proposition whereby it would seem feasible, within an early period, to attain interchange of licenses between states?

The replies received to date are as follows:

I have to state that, under the existing dental law of the District of Columbia, the Board of Dental Examiners is required to grant license to all graduates of all dental colleges which give a three year course of study, and to examine all other applicants, to test their qualifications, both in theory and practice of dentistry before granting license. We have a new bill before Congress, and, if enacted, the Board will have more latitude than it now has especially in respect to interchange of licenses between states.

In expressing my personal opinion upon the subject matter, will state that I fully indorse the suggestions made in your editorial, believing them a proper solution of the question.

C. W. APPLER,
Washington, D. C. President.

It is with the greatest pleasure that I accept your courteous invitation to give my views on Prof. Kirk's paper and the ideas advanced by yourself in the discussion of the same, and in your editorial in the February *Items of Interest.*

This subject has been one of grave consideration to me for a number of years, and I must allow that there are many alluring thoughts in both of the plans suggested, but, unfortunately, I cannot but see the other side of the picture.

As to the first suggestion, it would have to be made absolutely sure that in no case could there be connivance between the college and the pupil. There may be dishonest colleges, another writer in the same issue of the *Items of Interest* suggests that there are and that by the means you propose they will be forced to raise their standard. Admitting simply for the sake of argument that there may be some such dishonest colleges, what is the guarantee of the genuineness or originality of the answers to the college questions? If a professor in a college would offer to sell to a student up for graduation, the questions and answers of a State Board before whom he had to appear, might he not be willing to dispose of the answers to his own questions for the benefit of his own school, if he thought its reputation in danger? And after all, would this method be as thorough an "examination of the college" as by the present means? What is the difference whether the applicant is rejected on the college questions or the Board questions? Does it not reflect upon the institution in practically the same way?

As to the second suggestion, it is, in my opinion, theoretically most excellent, but practically impossible, as I do not believe that any means could be devised to prevent the questions from becoming marketable before some of the state examinations, owing to the differences in the dates of the college commencements, as well as the meetings of the State Boards.

Under the laws of New Jersey the manner of examining is left to the Board, so that in answer to your question on this subject I would say if our Board considered either of these suggestions practical, they could adopt it.

As to the last, of course we all have our ideas of Eutopia, but then along comes the other fellow to show they are not practical. I have my ideas on this subject; I have talked them and written them for years. In the first place, strange as it may sound, I am opposed to Examining Boards, not to the Boards themselves, understand, but the conditions which made such an impression necessary. When a graduate receives his diploma, he should receive with it the right to practice anywhere under the Stars and Stripes, without let or hindrance from any one.

How can this be accomplished ? Let the final examinations for graduation in all colleges be conducted by a National Board of Examiners, appointed by the government of the United States. A diploma issued under these circumstances would be universally received and the necessity of State Boards abolished. This may be a long way off, but, in my opinion, it is the only safe and practical solution of the problem, and with a combined effort from the profession, colleges, and State Boards, backed by the National Association, I believe it will succeed.

G. CARLETON BROWN, Secretary,
Elizabeth, N. J.

I have read Dr. Kirk's paper, also discussions, together with your editorial, very carefully, and must say the more I read them, the more confused I become in regard to the burning question. It is a question to which I have given much study and thought, and yet cannot say that I have arrived at any definite conclusion.

This Board is a member of the National Association of Dental Examiners, and as such is in full sympathy with its work—that of raising the preliminary standard of colleges. To my mind that is the most important question at this moment. What dentistry needs to-day, above all things, is an educated body of men, and this can only be accomplished by insisting upon the colleges admitting only educated men. This they must do. The time has arrived for it. If they do not acquiese willingly, then they must be either forced into line or else come to grief. This is the work of the National Association, and this is the issue it is pushing with all its might and main. This Board has taken a firm stand along this line, and is not recognizing diplomas from any college not on the recommended list. It has a bitter fight on its hands, but is going to win. The National Association having accomplished this, it will then, doubtless, take hold of the question of a "uniform standard of granting licenses throughout the states."

As our law requires us to grant licenses to all reputable graduates, we would not give you any suggestions as to the best manner of solving this question, so far as experience is concerned. I must say, however, that your idea is worthy of much consideration, and I believe comes as near solving the question as any that has thus far been offered, although I most certainly dissent from your opinion as to "State Boards being a necessary evil."

W. H. Carson, Sec'y,
Milwaukee, Wis.

As there is considerable diversity of opinion as to the feasibility of the suggestions in your communication, I am authorized to use my own discretion in answering.

Your first suggestion is not perfectly clear to me. If you mean that a list of the questions used at the college examinations be submitted to the Board, that could give us no idea of an applicant's status, and I am sure the college faculties would not allow inspection of answers.

The second suggestion, to have identical examination papers, would necessitate the holding of simultaneous examinations by the different State Boards. If one Board meets for examination one week, and another Board meets the following week, applicants might be enabled in some way to obtain a list of the questions.

Our law allows us considerable latitude, and any of the suggestions could be adopted, if feasible.

I am very sorry that we cannot give this matter more attention, but I assure you that our Board is in entire sympathy with any project that will elevate the standard of dentistry.

J. G. Heuisler, Sec'y.
Baltimore, Md.

It was impossible, after your letter came into my hands, to get our Board together in time to obtain an expression of the body. To myself, personally, either of the two propositions would be satisfactory, except that the first would, I fear, remove the test very little away from the faculties of instruction. This, you may deem, an uncharitable view to take of it, but so long as men's wings have not sprouted, safeguards appear necessary.

The use of identical questions, if feasible, would, to my mind, appear the most impartial. It would involve simultaneous examinations in all the states, just as two weeks ago, we, in this state, held such in three separate places.

As I interpret our law, there would be no necessity for further legislation for the adoption of either proposition in our state.

H. GERHART, Sec'y,
Lewisburg, Pa.

I am not in position to express the sentiments of the Board at this time, as our next Board meeting will convene on May 13.

To express an individual opinion at this time will say that considerable opportunity is afforded for discussion on either of the propositions presented. Both of the methods seem to have merit, and no doubt can be discussed at the National meeting.

J. H. SMYSER, Sec'y,
Chicago, Ill.

In reply to your communication will say that our Board does not meet again before the second Tuesday in July, and of course I can get no opinion of the Board.

Being a subscriber to *Items of Interest*, and holding the position I do on our Board, of course, I had already read carefully the articles referred to.

I would not object to your first suggestion, and do think, if carried out honestly, it would answer every purpose, but care should be taken that such papers be not tampered with by any one.

I have no objection to the second proposition, if the same care be taken to keep the questions well guarded by those who have the right to handle them.

It is a fact, according to my understanding, that our Board has discretionary power about examination, and could carry out the above propositions, if so desired.

You will notice that I seem particular about who should handle questions and examination papers. I have served on our State Board over ten years now, and have some experience about stolen questions and other underhand methods about examinations. Our Board is composed of graduates and has the reputation of being very strict, which may be the cause of my experience, and few members of the profession would believe the tricks employed by some to pass the examinations. Then, again, if some of the questions and answers I have seen in print are samples of the examination papers where some of the colleges grant degrees, I should not like to trust those same professors too far. So I say, if honestly carried out, your plan would be very well, but unless all parties who handle the papers can be trusted fully, we may better stick to the old way.

J. R. THOMPSON, Sec'y,
Newberry, S. C.

As our Board has not had a session since receiving your communication, what I have to say in reply represents my own ideas. But I know that these are shared in part by some of the other members of the Board.

Your first suggestion relative to applicants obtaining licenses upon the papers upon which their diplomas were granted is, I consider, a good one. At any rate it would afford a Board a better opportunity of knowing more of the qualifications of the applicant. To this, however, should be added a requirement of two days' practical work at the chair, and a license granted to applicant based on his standing in these. If the standing of State Dental Boards were uniform throughout the Union, then a license granted in our state should be respected in each and every State in the country. But such is not the case, for various reasons (in part, political) which cannot be discussed here. Even in a single State, the competency of the various Boards that come into power in a given number of years fluctuates.

Second suggestion of State Boards using identical examination questions prepared by a committee of the National Association of Dental Examiners, is a good suggestion also, provided the examining Boards meet at one and the same time throughout the country. This contemplates, of course, examining the product of our colleges. All this being equal, the better the material going into these institutions, the better the product.

If such a thing as having all dental colleges equally competent to give instruction to their students were possible, then the quality of the material entering into those institutions would alone need to be considered, and to a large extent we would be satisfied with the general output. But different motives govern different colleges. The same to a little less extent we find true with State Dental Boards

It has been suggested that the Dental Boards allow the colleges to accept any and all applicants and graduate whom they choose. That all State Boards examine all applicants for dental licenses with questions prepared by a committee of the National Association of Dental Examiners. That the answers be sent from each State (members of the N. A. D. E.) to the committee having prepared the questions, they to examine and grade them, and on the committee's markings, the State Board to either refuse or grant registrative certificate or license.

Our last State Legislature passed laws giving the Board greater power, and enables us to examine any or all applicants for license. I will send you a copy of the laws.

M. A. MASON, Secy,
Ft. Wayne, Ind.

Your first proposition about the granting of licenses to an applicant upon his original college examination papers, seems to me to be a very fair and just one, provided the said papers could be had direct from the colleges in an untampered condition.

Your second proposition meets with my hearty approval, and I hope to see the day when such a rule will be in force.

Third, yes. C. V. VIGNES, Sec'y,
New Orleans, La.

In regard to your inquiries concerning the granting and interchange of licenses, etc., I reply briefly as follows:

Our Board are unanimous in the opinion that some change is desirable, but they are not unanimous as to the practicability of any plan. We have had no meeting, but I have obtained an expression from all our members except one, and the second proposition, as stated in your letter, is regarded with favor, but one is not prepared to favor interchange of certificates.

I believe that it is the privilege of the Board to consider and adopt any of the propositions given.

I have not at present any propositions to make.

D. W. FELLOWS, Sec'y,
Portland, Me.

Your first proposition, if adopted as a whole, would defeat the very object for which it is proposed. Without going into the merits of the first clause, and admitting the desirability of its intent, it would be null and void if the second clause was adopted, for this reason. Immediately upon the graduation of a student he would present his papers to the local Board, receive his license, and the same would be accepted by all other Boards as satisfactory.

In place of this proposition, it seems to us that the plan adopted by the Nationrl Association of Dental Examiners is far preferable. Let each State Board have jurisdiction over the local colleges, enforce rules similar to those insisted upon by the National Association, seek means to compel their observance, and when the school comes up to the standard thus established, let all other Boards accept the diploma as fully meeting their requirements, and issue a license without further delay.

This rule of the National Association is strictly enforced by this Board, which is given such power by our law, and is found in practice to be equitable and just. We have had numerous applicants from graduates of colleges not recognized by the National Association, who, when notified that we could not give them a license under our law, have quietly departed without further trouble. The Board and the profession generally are much gratified at this provision of our law.

We are, therefore, of the opinion that proposition No. 1 is not feasible.

The second proposition meets with our hearty commendation. We will be glad to see such a provision adopted.

It is a fact that, without appeal to the legislature of our territory, either of the methods would be considered a satisfactory examination. The examination is left entirely to the discretion of the Board.

The suggestion given above seems to us, after six years' practical experience, to be nearer our ideas in regard to the interchange of licenses between the states than any method yet proposed.

D. W. MANLEY, Sec'y,
Sante Fe, New Mexico.

In regard to dental legislation, it seems to me that some of us have never comprehended that dental laws are police regulations, intended for the protection of the people, and for that alone.

The state cares nothing about protecting dentists from competition, but it cares a great deal about protecting the public from incompetent dentists. Nor does the state care where or how a dentist gets his education, provided, only that he has sufficient training not to prove dangerous to the community. Accordingly, in Kentucky, the legislature has constructed the State Board of Dental Examiners, solely for the purpose of protecting the citizens against incompetent dentists.

The law provides that no one shall practice dentistry in this state without a certificate of qualification from said Board, and that the evidence by which he may establish that he possesses sufficient learning and skill to entitle him to a certificate, shall be either such a diploma as proves the possession of the necessary attainments, or in the absence of such a diploma, the standing of an examination by the applicant by which he gives evidence of this fitness.

In a word, no one can practice without a certificate from the Board, and this can be secured by means of a diploma or an examination, and in no other way.

The Board deals wholly with individuals, and passes on the qualification of each individual applicant, as evidenced by his diploma or examination.

The Board owes no duties to any college, as a college; though, if the college maintains a standard of sufficient grade, its graduates are entitled to certificates upon presenting and filing their diplomas; but this is true, because, and only because, the diplomas taken as evidence, satisfy the Board that the parties holding them possess such knowledge and skill that they can safely be turned loose on the public.

The Board has no authority to require the applicant to produce his examination papers, and no right to issue any certificate on them; nor has the Board any right to recognize "licenses" granted by other states. Of course, a law might be passed authorizing the Kentucky Board to recognize licenses granted by other states, but the Board should not do so without such a law.

The prevailing notion that dental legislation is enacted, 1st, for the protection of the dentist from competition and 2nd, for the protection and benefit of the colleges, seems to me to be fraught with great danger to the cause of dental education. The protection of the people from incompetency is, and ought to be, the only ground on which such legislation can be maintained.

The great trouble in enacting satisfactory dental laws is the fact that a large majority of the people, both in and out of the calling, have no just conception of the scope of such laws.

We need education along these lines.

Of course, if a uniform law could be passed by all of the states, it would be very desirable; but, when we think of the difficulties in the way, it is an undertaking from which we may well shrink. We would first have to agree on what ideal law was, and then have it passed by all of the states exactly as agreed upon.

To any one who has had experience in trying to influence legislation, this proposition is absurd; the whole plan, to my mind, is simply impossible of accomplishment.

As to the proposition to have a National law regulating the whole matter, the advocates of such a plan evidently have no appreciation of the basis on which this government is formed. As mentioned in another part of this letter, all laws concerning the health of the people of the states come under the head of "police regulations;" these regulations begin and end with the state and cannot be made a matter of National law.

J. H. BALDWIN, Sec'y,
Louisville, Ky.

The idea of having an applicant for a license to practice dentistry in any state, present the examination papers upon which he was examined in college, is a good one, still this has its drawbacks, for it is a fact that quite a number of students are graduated each year, and from our best colleges, who are not qualified to practice dentistry. Where there are so many students, it is impossible for the college instructors to watch every student while doing the work required of him. For instance, a student (who has been a laboratory worker for several years) told me that during the past winter, he earned enough to pay nearly half of his expenses, helping fellow students do their deposing and practical cases required by the college. In mentioning this, I do not intend it as an attack on the colleges; far from that, but to show that some students manage to evade the requirements of the colleges.

2nd. I cannot favor the idea of having an identical list of questions gotten up by a committee of any association, for the reason that the various State Boards hold their examinations at different times. An applicant might be refused a license in one state, and being familiar with the list of questions, could go to another state and pass the examination. I would favor such a committee making up examination papers that would be uniform, but different questions for each state. Here, in New England, we have an Association of Dental Examiners, and we are trying to make our examinations uniform and to get uniform laws, that we may accept licenses from other states in place of an examination. I would like to see this done by all the states in the Union.

Our law says, "shall at its meetings, examine applicants, etc." If a license is given without examination we are discriminating. Law does not allow any one to discriminate. When the time comes for the recognition of licenses from other states, an amendment to the law, giving the authority to accept a license from another state in place of examination, can be easily obtained.

I hail the time when all states shall have such uniform laws and examinations, that the interchange of licenses may be brought about without doing an injustice to any one—colleges, examining boards, practitioners or patients.

GEORGE F. CHENEY, Sec'y,
St. Johnsbury, Vt.

In regard to your first proposition, I believe that such a method of examining candidates for state license would tend to encourage some colleges to fraudulent acts, and would tie the hands of the examiners, so that it would be impossible to stop it. I do not mean to say that all, or even a large per cent. of the colleges would take undue advantage of the examiners, but if even one did, that would be argument enough against the plans. This proposition carried to its last analysis is the same thing as registering diplomas straight out; the only difference is that one method has "red tape," and the other has not.

Your second proposition is a good one, but at this time I do not think it could be put into practice, without arousing political influences that would in some cases, injure the usefulness of the Board for years, and delay the interchange of licenses for some time to come. If this plan of examining candidates could be put in practice, we would not be far from interchange of licenses.

There is nothing in the Virginia law that would prevent a Board of Examiners from accepting either of the propositions mentioned.

I regret I cannot offer an alternate proposition to obtain interchange of licenses between states. An unrestrict- interchange of licenses between states, in my opinion, would not be desirable. An interchange whereby worthy and competent men could practice anywhere in the United States is desirable, and I would be glad to see the consummation of the plan by which this could be brought about. It will require time, careful thought, and considerable work to perfect a plan whereby justice can be done in the matter of interchange of licenses between states. Any plan should be thoroughly safeguarded against incompetent men.

H. W. CAMPBELL, Sec'y,
Suffolk, Va.

Personally, I have been pleased to read the advice, opinions and suggestions in the journal at your request. At present, I could not indorse either of the propositions contained in your letter, as being at all feasible or practical.

Our Board has long been interested in this matter, but realize that the attainment of even a portion of what is desired, will require a long time—legislative changes are not easily obtained everywhere—then we would not think it possible that the dental schools would consent to have their examination papers taken all about the country to be examined by a State Board at any and all times whenever a man desired to locate in its territory. Do not also think it feasible, for a time, to have the examinations throughout the country on the same day, and the issuing of questions left to the National Association of Dental Examiners.

Our Board, two years ago, organized with the other New England Boards, an association for the purposes under discussion; have held profitable meetings, and attained as much, I think, as we could in our work here. I think it is in evidence we cannot attain the objects the two propositions aim at, only in a comparatively slow manner. It will require a long time. The schools must be of even excellence, the Board of Examiners must have the same standard, provision must be provided to examine a candidate even if he has not attended a dental school, but I will not dwell longer upon my personal views.

G. E. MITCHELL, Sec'y,
Haverhill, Mass.

It is the unanimous opinion of the Massachusetts Board of Dental Examiners, that your first suggestion is impossible, for the reason that our twelve years' experience as an examining board has taught us that many of the young men who appear before us, recent graduates from reputable colleges, have utterly failed to pass our examinations, and are, in our opinion, incompetent to practice dentistry.

Your second proposition also seems to us very impracticable. It would necessitate examinations being held everywhere on the same day, which is not feasible, and would allow too much concentration of power in a few individuals.

Our New England Association of Dental Examiners was organized with a view to bringing about in New England, what seems to us the only practical way to interchange certificates, namely, a uniformity of legislation and of uniform standard of qualification. Though we have been in existence but two years, I feel we have made respectable progress; already two laws have been changed to harmonize with Massachusetts; and when those who are a little lax come into line, we hope to form a perfect law for New England.

J. F. DOWSLEY, President,
Boston, Mass.

Without going into discussion I would say, in regard to your first proposition, if all men were honest, if all were half as anxious to elevate the profession as they are to fill their pockets, this plan offers great inducements; but so long as the ungodly are in power, so long as colleges are run on a money-making basis, so long as there is a handsome surplus to divide among the favored few each year, I fear that plan would not be reliable.

Second Proposition—The first thing should be a uniform standard for matriculation, and this should not be a farce, but should be placed high enough. so that our eyes and ears will not be constantly shocked by bad chirography and worse grammar. If you could sit with an examining board for one session, and see the time consumed by trying to read writing that would make a Chinaman turn green with envy, grammar that is execrable, and spelling that gave you "bad spells," you would think and say at once, "For Heaven's sake, make the standard high enough that the people be not shocked by such display of ignorance."

This may seem overdrawn, but quite a percentage of that class come up every year, and it is needless to say they do not favor raising the standard.

For our state, we have settled upon a full High School course, as a prerequisite for matriculation. This insures a fair English education. Let the other states adopt the same standard, or one equally as good, and we are all on a par to start with.

Then let a committee be appointed by the National Association, National Board of Examiners, State Societies or by such power as may be deemed best, to formulate questions for examination. Let all State Boards adopt these questions and we have a uniform examination throughout the states, and a license in one state would be equally good in another. Of course, the applicant must have taken his degree in regular form.

I think the New York State Board of Dental Examiners, in connection with the Regents of the University, have full power without further legislation to adopt any method of examination which may be deemed best for the elevation of the dental profession.

FRANK FRENCH, Sec'y,
Rochester, N. Y.

None of the members of the Board seemed to altogether approve of either the first or second proposition. There is such a different standard of qualification in different sections of the country, that it is very difficult to fix one to suit all. What might suit the standard of West Virginia, might be very far from the standard of New York or Boston. Colleges in different part of the country are turning out handsomely finished, full fledged, first class dentists by the hundred—yes, by the thousands—with beautifully finished diplomas, on which very few are able to read anything except their own names. Many of them are as well qualified to practice dentistry as a hog to teach Latin. Some are bright boys, and, probably, are good mechanics, so after some practice and experience may make good mechanical dentists.

We think as a Board, we have the right to determine the qualifications, and decide the question whether or not the candidates are qualified and fit to practice dentistry in our section of the country. A Board of Dental Examiners in Boston, and possibly in New York, might give their noses a celestial turn if a West Virginia Board should attempt to indicate to them what sort of an examination they should give their candidates.

It certainly would be a great convenience, if some universal law could be made, that would suit all the different states.

I have not gray matter enough to work out so important a question. It is a problem requiring the head of an experienced legislator.

We think we are all practical sort of men, and would be pleased to vote for anything that seems feasible, and would be glad to have some universal law that would make one examination do for all the different states.

CHAS. H. BARTLETT, President,
Parkersburg, W. Va.

I am convinced that we need a uniform standard of education. Either of your plans would accomplish the purpose, but my preference would be for the second proposition.

I think that under the law of this state, the examination is left to the determination of our Board, and we would be at liberty to carry out either of the proposition.

R. H. JONES, Sec'y,
Winston, N. C.

I am of the opinion that the second proposition suggested is the more desirable. I think it should not be limited though, to Boards which are members of the National Association. I do not believe in making it compulsory for Boards of separate states to join the association. Think it is a step in the right direction.

R. A. SHINE, Sec'y,
Tallahassee, Fla.

In regard to the proposition that State Boards should issue a license upon presentation of the diploma and the original college examination papers as guarantee of the knowledge of the applicant for examination, I would say, that it seems to me that this is almost an impossibility, and, in fact, would be so in many cases; especially the men who are now graduates and who cannot obtain papers written by them when in college, and even if they could secure their old individual examination papers, it is a question whether they would be of such character as to meet with the approval of the State Boards at the present time.

The suggestion that the State Boards agree to a definite examination by questions issued by a Committee of the National Board of Dental Examiners, I think is the only fair solution of the question—with this proviso; that where it is possible, the State Boards agree to the same date for the examination in the different states, say during January and July of each year, and within, if possible, the same week of these months, at which time all applicants for examination should be given the questions as issued from a Committee of the National Board of Dental Examiners. These questions to be furnished to the State Boards in such manner that no knowledge of them could be obtained by any person or persons who are to be examined. Papers written by parties examined under these conditions to become a part of the evidence of their ability as dental practitioners. These papers with a license from any State Board, issued under the above conditions, to be accepted by all other State Boards agreeing or being parties to this agreement. This arrangement would allow the practitioner to take the State Board examinations at any of these semi-annual meetings, and after having once passed any State Board or the National Board of Dental Examiners' questions, he would be eligible for license in any other state agreeing to this arrangement, no matter at what time he might desire to change his residence.

I think the greater part of the State Boards who are members of the National Board of Dental Examiners would permit of this arrangement, and to so change the date of their meetings by which we could have a uniform date, say, the second week in July and January, at which time all State Boards would hold a meeting, and each and every applicant for license would be compelled to have the same examination, thereby showing no favoritism of politics to any candidate.

It is almost impossible for State Boards to agree to accept the diploma without question, from any college. Mistakes will happen, and men go through by some method or other, who are thoroughly unfit to practice dentistry or anything else.

The North Dakota Board finds the only successful rule, "To examine all candidates without discrimination as to college or other conditions." After some eight or ten years knowledge of the Board's workings, I believe that this is the only one fair method of arriving at the definite understanding of the knowledge of the applicant for license. In fact, we have had to turn down men from some of the best colleges, while others from colleges of less note have proven in every way, so far as examination would demonstrate, much better prepared for practice.

H. L. STARLING, Sec'y,
Fargo, N. D.

Our Board has had no meeting recently, and I cannot therefore send you the opinion of this Board regarding interchangeable licenses. My own judgment is that the second proposition would come nearest to being practical in its workings. Our law defines thirteen branches, including practical demonstration of ability, that the applicant must pass in.

If these were covered by an examination in another state, it might be legal to consider this a satisfactory examination, but of course, this is a point purely to be decided by the Board.

W. E. BURKHART, Sec'y,
Tacoma, Wash.

In regard to suggestion No. 1, the idea of having applicants present to State Boards their original papers upon which they were examined is to me commendable, in so far that it assists the Dental Boasd, but the granting of a license upon said papers and the interchange of licenses granted upon such evidence of ability, would never meet with my approval.

Suggestion No. 2—State Board making use of identical examination papers; this is commendable, provided, however, they be the original papers and not made interchangeable; your method, however, would meet with no opposition from our legislative body because our laws are founded upon findings from the National Association of Dental Examiners.

I have no propositions to make in regard to making licenses interchangeable, for it will certainly meet with opposition here.

GEO. E. ELLERBECK, Sec'y,
Salt Lake City, Utah.

Thus, gentlemen, we have before us in concrete form, an expression of opinion from twenty-one Examining Boards, a summary of which brings out some very important facts, not the least of which is the fear that dishonesty would militate against any proposition. Five states, apparently speaking from experience, suggest that dishonesty is traceable to the colleges direct, while some believe that the greatesl care is requisite to prevent fraudulent possession of examination questions by applicants for license. If these accusations are well founded, it is a serious blot upon the honor of those in charge of our educational system. Further consideration of this feature is not requisite here, as we are merely seeking to achieve interchange of license, and if there be dishonesty, licenses should not be procurable, even in one state.

To those who have believed that an interchange of license is a dream of Eutopia, the present series of letters must prove as encouraging, as they will be surprising. Two propositions were considered. The first provides that the candidate shall present his college examination papers which shall entitle him to a license, if satisfactory to the Examining Board. To this proposition four States assent, viz., District of Columbia, South Carolina, Indiana and Louisiana, while five others approve the scheme while doubting its possible enactment. These are Wisconsin, Pennsylvania, Illinois, Vermont and North Carolina. Maine, Washington and Florida merely express a preference for the alternate proposition, so that their vote is neither for nor against. Nine States, however, oppose the idea, these being New Jersey, New Mexico, Kentucky, Virgtnia, Massachusetts, New York, West Virginia, North Dakota and Utah.

There being nine States that oppose the proposition, and only nine in favor of it, the majority of which latter are doubtful, we may dismiss the idea as untenable, especially as its perfect operation would require the co-operation of the colleges, which could be procured, but only by force. Many of the objections which have been made can be answered, several being due to misapprehension of the scheme, but as we abandon the idea it is futile to waste time in further discussion.

The second proposition has met with such a reception that it merits our most studious and earnest consideration. Fourteen states approve the plan, only three being opposed. Of the others, two failed to approve, merely because they preferred the first proposition; they would certainly accede to the second. Another, New Jersey, thinks the scheme excellent, but impracticable, while a fourth, Vermont, opposes only the feature of identical questions, and suggests a modification which could well be adopted. Thus three states oppose, Kentucky, Massachusetts and West Virginia, while seventeen indorse the idea, these being District of Columbia, New Jersey, Wisconsin. Pennsylvania, Illinois, South Carolina, Indiana, Louisiana, New Mexico, Virginia, Vermont, New York, North Carolina, Florida, Washington, North Dakota and Utah.

The next matter of moment is the third question, whether the Examining Boards being willing to adopt the plan, have they the legal power ? Every state admits that such power exists without further legislation, except Kentucky and Vermont, but we are informed that in Vermont, an amendment permitting interchange of license on an equable basis could be obtained.

The greatest difficulty in connection with all schemes heretofore proposed, has been the impossibility of obtaining further legislation throughout the States. I am exceedingly proud to have evolved a method, agreeable to the examiners of seventeen states, which can be put into operation without special legislation; a plan therefore within the scope of existing laws, and for that reason, worthy, I think, of adoption and trial for a time at least.

Certain objections have been brought out, but these can certainly be met. The greatest trouble seems to be that in order to avoid dishonesty, it would be requisite that the examination should be held simultaneously. One correspondent says this is impossible, but he does not explain why. So far as the examiners are concerned, they certainly should be able to adopt any date agreeable to the majority of those coming into the agreement. The only difficulty which occurs to myself seems to be that students of some colleges would be compelled to lose some months perhaps after graduation before license could be obtained. But this only adds a brief period to their studentship at worst, and if this were thought to be inequitable such graduates might be permitted to practice undisturbed until the date of the first examination after their graduation. If the examinations were held semi-annually this would be but a comparatively brief period.

The correspondent from Vermont objects to identical examination questions for all states, and suggests a single committee authorized to arrange the questions, but having power to promulgate separate questions for different states.

After due consideration of all the letters received I have the honor to suggest the following, as a solution of the vexed problem :

First—That a single National Committee shall arrange the examination questions for use by the State Examining Boards and that license granted by one state, a party to this agreement, shall entitle the holder to practice in any of the states so agreeing.

Second—That identical questions shall be used by those states which could hold examinations simultaneously, but that the committee shall provide separate sets of questions, of equal grade, for such states as could not agree to simultaneous examinations.

Third—Each State Board, in addition to using the National Committee's questions for the theoretical examinations, the successful candidate. however, not being compelled to pass another such examination when presenting his license to another state.

Fourth—That each state, party to this agreement, shall pay a stated fee for its set of questions, the fee being higher where special questions are provided, the sum thus raised to be a remuneration to the committee for the arduous labors which would be entailed by this plan.

This ends my report, gentlemen, but before closing, I beg that the opportunity now afforded of inaugurating a movement which may bring us a solution of this problem, be not lost. May I ask the Society to take some definite action upon the proposition here expounded ? If the final plan here set forth seems adequate, will the Society so vote, and so voting will they send a communication upon the subject to the National Association of Dental Examiners and to our National Association, urging a trial of the plan ?—*Items of Interest.*

